# A Framework for Linear and Non-Linear Registration of Diffusion-Weighted MRIs Using Angular Interpolation

**DOI:** 10.3389/fnins.2013.00041

**Published:** 2013-04-04

**Authors:** Julio M. Duarte-Carvajalino, Guillermo Sapiro, Noam Harel, Christophe Lenglet

**Affiliations:** ^1^Department of Radiology, Center for Magnetic Resonance Research, University of Minnesota Medical SchoolMinneapolis, MN, USA; ^2^Electrical and Computer Engineering, Computer Science, and Biomedical Engineering, Duke UniversityDurham, NC, USA

**Keywords:** angular interpolation, diffusion, fiber orientation, registration, tensor

## Abstract

Registration of diffusion-weighted magnetic resonance images (DW-MRIs) is a key step for population studies, or construction of brain atlases, among other important tasks. Given the high dimensionality of the data, registration is usually performed by relying on scalar representative images, such as the fractional anisotropy (FA) and non-diffusion-weighted (b0) images, thereby ignoring much of the directional information conveyed by DW-MR datasets itself. Alternatively, model-based registration algorithms have been proposed to exploit information on the preferred fiber orientation(s) at each voxel. Models such as the diffusion tensor or orientation distribution function (ODF) have been used for this purpose. Tensor-based registration methods rely on a model that does not completely capture the information contained in DW-MRIs, and largely depends on the accurate estimation of tensors. ODF-based approaches are more recent and computationally challenging, but also better describe complex fiber configurations thereby potentially improving the accuracy of DW-MRI registration. A new algorithm based on angular interpolation of the diffusion-weighted volumes was proposed for affine registration, and does not rely on any specific local diffusion model. In this work, we first extensively compare the performance of registration algorithms based on (i) angular interpolation, (ii) non-diffusion-weighted scalar volume (b0), and (iii) diffusion tensor image (DTI). Moreover, we generalize the concept of angular interpolation (AI) to non-linear image registration, and implement it in the FMRIB Software Library (FSL). We demonstrate that AI registration of DW-MRIs is a powerful alternative to volume and tensor-based approaches. In particular, we show that AI improves the registration accuracy in many cases over existing state-of-the-art algorithms, while providing registered raw DW-MRI data, which can be used for any subsequent analysis.

## 1 Introduction

Diffusion-weighted magnetic resonance imaging (DW-MRI) is a non-invasive imaging technique that measures the diffusion of water molecules in biological tissues, such as the brain’s white matter, along several gradient directions. This information can be used to estimate the local orientation of fiber bundles, since water diffusion is favored along the fiber orientation, providing critical information for neuroscience and clinical studies (Jones et al., 1999; Basser and Jones, 2002; Filler, 2009).

Registration of DW-MRIs is a key step in population studies or brain atlases construction, among other important tasks. However, registration of such data is more challenging than for three-dimensional scalar images, not only because of its high dimensionality (hundreds of volumes) or noise and artifacts present in MR scans (Jones et al., 1999; Behrens et al., 2003; Le Bihan et al., 2006; Stobbe and Beaulieu, 2011), but because the signal, and any subsequently estimated local fiber orientation model, must remain consistent with the underlying fiber geometry after image transformations (Zhang et al., 2006; Barmpoutis et al., 2007; Cheng et al., 2009; Dhollander et al., 2010; Verma and Bloy, 2010; Yap et al., 2010; Du et al., 2011; Geng et al., 2011; Raffelt et al., 2011).

The simplest approach to DW-MRI registration consists in registering these datasets using a single transformation obtained from a representative volume, such as the Fractional Anisotropy (FA) (Pierpaoli and Basser, 1996), or the non-diffusion-weighted (b0) image. However, DW-MR registration using a single volume can be unsatisfactory, since it disregards the fiber orientation information provided by the diffusion-weighted volumes.

Another approach consists in registering instead the DTI estimated from the DW-MRI using the Stejskal and Tanner (1965) equation and assuming that the diffusion process follows a Gaussian distribution (Basser et al., 1994). Besides the spatial registration of DTI data, the images must also be reoriented so as to be consistent with the transformations made to the anatomy (Alexander et al., 2001; Zhang et al., 2006).

In order to overcome known limitations of the diffusion tensor model (Skare et al., 2000; Descoteaux et al., 2006; Zhang et al., 2006; Barmpoutis et al., 2007; Hess and Mukherjee, 2007; Koay et al., 2009), higher order models have been proposed (Barmpoutis et al., 2007; Cheng et al., 2009; Dhollander et al., 2010; Verma and Bloy, 2010; Yap et al., 2010; Du et al., 2011; Geng et al., 2011; Raffelt et al., 2011). Nevertheless, relying on such diffusion models might not completely capture the information contained in the raw data and could therefore affect the registration accuracy. In addition, these models are not generic and rely on specific assumptions.

The concept of “Angular Interpolation” (AI) was proposed by Tao and Miller (2006) to perform linear registration of the raw DW-MRIs, without imposing any specific diffusion model. The spatial registration with an angular interpolation correction of the image intensities attempts to match the registration of the underlying fiber structures. The evaluation of AI was entirely qualitative in Tao and Miller (2006). In this work, we extend the AI algorithm to non-linear registration and perform a wide range of tests that include human brain DW-MRIs undergoing known synthetic linear and non-linear transformations. We also evaluate our algorithm on pairs of human brain DW-MRIs to quantify the registration accuracy of AI versus b0-based registration (FLIRT^1^ and FNIRT^2^ in FSL^3^), and two well-known libraries that support registration of DTIs: DTI-TK^4^ (Zhang et al., 2006) and MedINRIA^5^. In addition, we provide an efficient C++ implementation of the algorithms in FSL, an open source and publicly available library that provides several analysis tools for FMRI, MRI, and diffusion MRI brain imaging data. Our implementation also handles the registration of DW-MRI datasets obtained with different gradient tables.

A recent work (Wang et al., 2011) compares the registration accuracy of several state of the art non-linear registration algorithms: FNIRT, MedINRIA, DTI-TK, and several demons algorithms using real brain images and an atlas. The conclusions of this work were that DTI-TK has the best registration performance. We will demonstrate that AI has a competitive performance, while providing registration of the raw DW-MRI data, not just the tensors.

Some other works have been recently published addressing the issue of gradient reorientation of DW-MRIs, in the context of image registration (Dhollander et al., 2010; Yap et al., 2010), and atlas building (Bouix et al., 2010). In particular, Yap et al. (2010) compute rotated gradient directions to estimate the rotated tensors from the spatially registered DW-MRIs. Dhollander et al. (2010) and Bouix et al. (2010) model the DW-MR datasets as a weighted superposition of spherical harmonic polynomials, updating the intensity of the registered DW-MRIs with the estimated weights and affine transformed basis. In fact, Dhollander et al. (2010) shows, using a synthetic example, that AI can produce undesirable rotation effects on fibers that have suffered pure shearing. This is a well-known problem in DTI registration (Alexander et al., 2001): pure shearing contains a complex rotational component not accounted for, when extracting the rigid rotational component of the affine matrix. In order to account for the complex rotation effect that shearing, stretching, or non-uniform scaling can have on the fiber orientation, Alexander et al. (2001) proposed the preservation of principal direction (PPD) algorithm. Dhollander et al. (2010) work can be seen as an extension of the PPD algorithm to their model of DW-MR datasets. However, as Zhang et al. (2006) and Alexander et al. (2001) indicate, there is little difference between using PPD or pure rigid rotation to register real DT-MR images and that is why it has been successfully used in the past (Zhang et al., 2006; Thomas Yeo et al., 2009), with a much lower computational cost.

Based on the previous considerations, we choose in this work to use the pure rigid rotational component of the affine matrices^6^. Nevertheless, AI can also be implemented using PPD to extract the rotation of general affine transformations to rotate independently the gradients in the image, making it similar to the work of Dhollander et al. (2010) and Bouix et al. (2010), with the advantage of not having to model the image as a superposition of polynomials.

The main contributions of this work are:

- The extension of AI to non-linear registration (Section 2.2).
- Improved computational performance of AI and quality of the associated FA estimate (see Section 2 in Appendix).
- Handling of DW-MR images with different gradient tables (Sections 2.1, 2.3).
- Comparative study of AI versus b0-based registration and two well-known tensor-based registration methods, MedINRIA and DTI-TK (Section 2.3).
- Public domain software to run AI within FSL^7^, including AI correction to b0-based and DTI-based spatial only registration. This code will be released upon publication as a plug-in for FSL^8^.

The concept of angular interpolation for linear registration is introduced in Section 2.1. In Section 2.2, we extend AI to non-linear registration by extending the FNIRT model to register diffusion-weighted MR images using AI. Some important details on the implementation of the registration algorithms in FSL are provided in the Appendix. The experiments performed, as well as the images used in this work are presented in Section 2.3. The results of these experiments are presented and discussed in Section 3. We present the conclusions of this work in Section 4. Finally, note that the Appendix includes more details about the implementation and complementary figures.

## 2 Materials and Methods

### 2.1 Affine registration with angular interpolation

For an *n*-voxel DW-MRI dataset taken with *m* different gradient directions, the MR attenuation due to water diffusion in organized tissues (such as the brain white matter) can be modeled as (Stejskal and Tanner, 1965; Basser and Pierpaoli, 1996; Jones et al., 1999; Hasan et al., 2001)

(1) $$S\left(x_{i},g_{j},\tau \right)=S_{0}\int_{{\mathbb{R}}^{3}}P\left(x_{i}+r|\tau \right)e^{-2\pi \text{i}q_{j}\cdot r}dr,$$

where, $x_{i}\in {\mathbb{R}}^{3},\;i=1,\;\ldots ,\;n$ are the discrete spatial samples, $g_{j}\in {\mathbb{R}}^{3},\;j=1,\;\ldots ,\;m$, are the diffusion gradients, *τ* the molecular diffusion time, *S*_0_ the anatomical image taken with no gradient applied, **q***_j_* = γδ**g***_j_/*2π is the displacement reciprocal vector (with γ the gyromagnetic ratio of water protons and δ the duration of the diffusion gradients), **r** the displacement vector relative to **x***_i_*, (·) is the dot product between vectors, and *P*(**x***_i_* + **r**|*τ*) the ensemble average propagator (EAP) at **x***_i_*, whose shape depends on the underlying fiber bundle structures. Without any loss of generality, we can consider that ||**g***_j_*||_2_ = 1, hence from (1), the DW-MRI signal can be seen as samples of the Fourier transform of the EAP taken along different gradient directions on the unit sphere. Hence, the intensity *I*(**x***_i_*, **g***_j_*) of the DW-MRI is a function of the position, gradient orientation, and the underlying fiber structure (given by the EAP at each voxel).

From now on, we assume that the volumes of the DW-MR datasets have been properly aligned to correct for possible mismatches (Section 2.3.1), due to eddy current distortions, geometric distortions, and head motion during the data acquisition. Hence, it is assumed here that the DW-MRI datasets measure the true underlying three-dimensional structures (through diffusion), and any linear or non-linear spatial transformation should be applied to every volume in the data set to reflect the corresponding spatial transformation of those structures.

Let *I_ref_*(**x***_i_*, **g***_j_*) and *I_test_*(**x***_i_*, **g***_j_*) be the reference and test DW-MRIs, respectively. Also, let **A** be a given transformation matrix that registers the test to the reference DW-MRI. The spatial registration of the test DW-MRI would be ${Ĩ}_{test}\left(A^{-1}{\text{x}}_{i},\;{\text{g}}_{j}\right),$ requiring interpolation^9^ to compute the intensity at the spatial coordinates **A**^−1^**x***_i_*. Hence, all the *m*-volumes in the DW-MRI can be spatially registered using the same affine registration. However, performing only spatial registration ignores the fact that the underlying fibers have changed their orientation with respect to the gradient orientations **g***_j_* and hence, in general, the intensity of the reference image will not be the same as the intensity obtained after spatial registration of the test image.

The most widely used reorientation strategy in DTI consists in reorienting the tensors as **RDR***^T^* (Alexander et al., 2001; Zhang et al., 2006), where **D** is the second order diffusion tensor and **R** is the orthonormal matrix corresponding to the rotational component of **A**. The rotational component of a matrix can be obtained using the Finite Strain (FS) method, based on the polar decomposition (Higham, 1986), **A** = **RS**, where **S** is the strain component of **A**. A more accurate estimation of the rotational component of **A** can be obtained with the preservation of principal direction (PPD) algorithm, proposed by Alexander et al. (2001). Here, and as in Zhang et al. (2006), we use the FS method given its good performance by comparison with PPD, when the images are real DW-MRIs (Alexander et al., 2001; Zhang et al., 2006). Also, FS provides an analytical decomposition of **A** that allows the use of closed form derivatives, necessary when using gradient descent minimization methods (as in FNIRT), and it is simpler and computationally more efficient than PPD. We must point out here that in the original paper on AI (Tao and Miller, 2006), the authors do not use the rotational component of **A**, but use instead **Ag***/*||**Ag**||_2_ to modify the gradient directions (see Section 2.1). However, as Alexander et al. (2001) indicate, the shape of regions in the image can change (due to spatial registration), but the underlying tissue microstructure can only change through pure rotation.

We will explain now what angular interpolation is. From the fact that ${\text{g}}_{j}\cdot \left(\text{Rr}\right)=\left({\text{R}}^{T}{\text{g}}_{j}\right)\cdot \text{r}$ and (1), we can see that sampling the reoriented fibers using the gradients **g***_j_* is equivalent to sampling the unrotated fibers using the rotated gradients ${\text{R}}^{T}{\text{g}}_{j}$. Hence, the key idea in AI is that under an affine transformation, the rotation of the underlying fiber orientations can be obtained by rotating the applied gradient ${\text{g}}_{j}^{\prime }={\text{R}}^{T}{\text{g}}_{j}$, so that in the new coordinate system provided by ${\text{g}}_{j}^{\prime }$, the underlying fiber orientations are rotated by **R**, as desired. This seems trivial, but the advantage of using AI is that we do not need to compute tensors from the (noisy) images or assume any other diffusion model. Also, we do not need to know *a priori* the fiber orientations, or if there are fiber crossings on a given voxel. We simply rotate the known gradients as ${\text{R}}^{T}{\text{g}}_{j}$. and compute, by angular interpolation, the DW-MRI corresponding to those new orientations, based on the full original test DW-MRI dataset. Notice here that AI is done on the unit sphere (||**g***_j_*||_2_ = ||**R***^T^***g***_j_*||_2_ = 1) and that is why it is called angular interpolation. Notice also that AI does not spatially affect the intensity of the volumes, it interpolates among the volumes corresponding to the known gradient directions **g***_j_*. Hence, AI provides a natural way to register DW-MR images. The usual spatial interpolation is complemented by angular interpolation to reflect the change in intensity due to fiber reorientation.

In practice, we do not have an oracle that provide us with the optimal affine transformation to register the test image. We need to estimate the affine matrix by minimizing a cost function that measures the distance between the reference and registered test images. Hence, we want to find the optimal affine transformation **A** that minimize the cost function,

(2) $$C\left(A\right)=\overset{n}{\underset{i=1}{\sum }}\overset{m}{\underset{j=1}{\sum }}𝒟\left(I_{ref}\left(x_{i},g_{j}\right),{Ĩ}_{test}\left(A^{-1}x_{i},R^{T}g_{j}\right)\right),$$

where, *D* is an appropriate distance metric, such as Euclidean distance, cross-correlation, or Mutual Information.

In general, angular interpolation of DW-MR images can be done as (Tao and Miller, 2006),

(3) $$\dot{I}\left(x_{i},g_{j}\right)=\frac{\sum_{k=1}^{p}\alpha_{k}I\left(x_{i},g_{k}\right)}{\sum_{k=1}^{p}\alpha_{k}},\;i=1,\ldots ,n,\;j=1,\ldots ,m,$$

where α*_k_* are the interpolation weights assigned to each of the *p* volumes in the test image, defined as non-linear functions of $cos^{-1}\left({\text{g}}_{j}\cdot {\text{g}}_{k}\right)$, the geodesic distance on the unit sphere between the desired target gradient, **g***_j_*, and the available gradients in the image, **g***_k_* (see Tao and Miller (2006) for details on the non-linear function used). Since **g***_j_* can be any gradient direction, we can use AI to match two DW-MR images with different gradient tables, by defining **g***_j_* as the gradients in the reference image and **g***_k_* the available gradients in the test image. From now on, we assume that the gradients in the test image have been matched to the gradients in the reference image (using AI) and hence, *p* = *m*.

The linear registration algorithm in FSL is FLIRT (Jenkinson et al., 2002), which provides single volume registration using Euclidean distance, Cross-correlation, and Mutual Information metrics. Our extension of FLIRT to register DW-MRIs is based on AI, where, besides simultaneous spatial registration of all the volumes, AI is also applied on all gradient directions, at each step of the minimization, as indicated in (3), where can be any of the distance metrics provided by FLIRT. We will explain in much more detail the extension to non-linear registration in the next section, since there, the implementation is more involved with the particular cost function and small displacement model used in FSL.

### 2.2 Non-linear registration with angular interpolation

In the previous section, we introduced the concept of angular interpolation to linearly register DW-MRIs. This idea can be extended to non-linear registration of DW-MRIs, if the non-linear transformation is locally affine. Let *T*(**x**) = **x** + **u**(**x**) be a non-linear transformation applied at **x** ∈ ℛ^3^, where **u**(**x**) is the so called deformation field, then (Irfanoglu et al., 2008),

(4) $$\begin{array}{llll} T\left(x\right) & =x+u\left(x\right)=A\left(x\right)x+t\left(x\right), & & \\ A\left(x\right) & =I+J_{u}\left(x\right),\;J_{u}\left(x\right)=\frac{du\left(x\right)}{dx}, & \end{array}$$

where, **A**(**x**) is the equivalent local affine transformation at the spatial coordinate **x**, **t**(**x**) a pure translation vector, **I** the identity matrix, and **J_u_** the Jacobian of **u**. Hence, the deformation field can be decomposed into a local affine transformation plus a translation field.

The non-linear registration tool in FSL is FNIRT (Andersson et al., 2007). However, FNIRT can only be used to register scalar volumes^10^ and it works under the assumption of a small deformation field. Here, we will extend FNIRT to the non-linear registration of DW-MRIs using angular interpolation (AI). We extend here FNIRT, based on the derivation made by Andersson et al. (2007), for the case of scalar volumes. In this section, we will refer only to our FNIRT-based AI model and hence, the equations apply to DW-MRIs, not just single volumes. As in the case of affine registration, where the affine matrix applies to all the volumes in the image, the deformation field here is three-dimensional and also applies to the whole DW-MR image.

FNIRT models the deformation field as a linear superposition of three-dimensional cubic B-splines (Andersson et al., 2007),

(5) $$u\left(x_{i}\right)=\underset{k}{\sum }w_{k}B_{k}\left(x_{i}\right),\;k=1,\;\ldots ,\;s,$$

where **w***_k_* is the weight along each coordinate for the B-spline *B_k_*, and *s* the number of splines, which depends on the spatial resolution of the field. Then, the non-linearly warped test image ${Ĩ}_{test}\left({\text{x}}_{i}+\text{u}\left({\text{x}}_{i}\right),{\text{g}}_{j}\right)={Ĩ}_{test}\left({\text{x}}_{i},\;{\text{g}}_{j},\;\text{w}\right)$ is a function of the position, the gradient orientations, and **w**, the vector of all the *B*-spline coefficients defining the deformation field. Notice that all the diffusion images are in ℛ*^m × n^*, where *n* correspond to the number of voxels in the image and *m* the number of diffusion gradients. Hence, the entire DWI series can be expressed in vector form as

(6) $$\begin{array}{c} I_{ref}={\left[I_{ref}^{T}\left(g_{1}\right)\;\ldots I_{ref}^{T}\left(g_{m}\right)\right]}^{T}\in {\mathbb{R}}^{m\times n},\\ {\tilde{\text{I}}}_{test}\left(w\right)={\left[{\tilde{\text{I}}}_{test}^{T}\left(g_{1},w\right)\;\ldots {\tilde{\text{I}}}_{test}^{T}\left(g_{m},w\right)\right]}^{T}\in {\mathbb{R}}^{m\times n}, \end{array}$$

where

$$\begin{array}{llll} I_{ref}\left(g_{i}\right) & ={\left[I_{ref}\left(x_{1},g_{i}\right)\;\ldots I_{ref}\left(x_{n},g_{i}\right)\right]}^{T}\in {\mathbb{R}}^{n}, & & \\ {\tilde{\text{I}}}_{test}\left(g_{i},w\right) & ={\left[{Ĩ}_{test}\left(x_{1},g_{i},w\right)\;\ldots {Ĩ}_{test}\left(x_{n},g_{i},w\right)\right]}^{T}\in {\mathbb{R}}^{n}. & & \end{array}$$

The cost function to be minimized in FNIRT is the mean square error^11^

(7) $$C\left(w\right)=\frac{1}{mn}E^{T}E,E={\tilde{\text{I}}}_{test}\left(w\right)-I_{ref},$$

where **E** is the error between the reference and test images. We are interested in estimating the deformation field, defined by the coefficients **w**, such that it minimizes *C*(**w**).

We are going to derive next a solution to (7) that extends the equations derived in (Andersson et al., 2007) for single volume registration to registering diffusion data sets. FNIRT solves (7) using a Gauss-Newton minimization method,

(8) $$w^{l+1}=H{\left(w^{l}\right)}^{-1}\;\nabla C\left(w_{l}\right),\in {\mathbb{R}}^{s}$$

where *l* is the iteration index and **H** is the Hessian, which can be approximated in terms of the Jacobian as $H\left(w^{k}\right)\approx \frac{2}{mn}J{\left(w^{k}\right)}^{T}J\left(w^{k}\right)$ (Fletcher, 1987), with the Jacobian given by

(9) $$\begin{array}{llll} J\left(w\right) & =\frac{dE}{dw}=\frac{d\left({\tilde{\text{I}}}_{test}\left(w\right)\right)}{dw} & & \\ & ={\left[\frac{d\left({\tilde{\text{I}}}_{test}^{T}\left(g_{1},w\right)\right)}{dw}\ldots \frac{d\left({\tilde{\text{I}}}_{test}^{T}\left(g_{m},w\right)\right)}{dw}\right]}^{T}\in {\mathbb{R}}^{m\left(n\times s\right)}. & \end{array}$$

Let $x^{\prime }=x+u\left(w\right)$,

(10) $$\frac{d\left({\tilde{\text{I}}}_{test}\left(g_{j},w\right)\right)}{dw}={\left[\frac{\partial {Ĩ}_{test}\left({x^{\prime }}_{i},g_{j}\right)}{\partial w_{k}}\right]}_{ik}=J^{j}\left(w\right)\in {\mathbb{R}}^{n\times s}.$$

By the chain rule and (5),

(11) $$\begin{array}{llll} J^{j}\left(w\right) & ={\left[\frac{\partial {Ĩ}_{test}\left(x^{\prime },g_{j}\right)}{\partial x^{\prime }}{\left|\right.}_{{x^{\prime }}_{i}}⊙\frac{\partial x^{\prime }}{\partial w_{k}}{\left|\right.}_{x_{i}}\right]}_{ik} & & \\ & ={\left[\frac{\partial {Ĩ}_{test}\left(x,g_{j}\right)}{\partial x}{\left|\right.}_{{x^{\prime }}_{i}}B_{k}\left({x^{\prime }}_{i}\right)\right]}_{ik}, & \end{array}$$

where ⊙ is the Hadamard product and $\frac{d\left({\tilde{\text{I}}}_{test}\left(g_{j},w\right)\right)}{dw}$ corresponds to the gradient of the moving image being warped by the deformation field, conveniently expressed as a vector derivative to highlight the derivative chain rule, and following the derivation in Andersson et al. (2007). This gradient is computed in FNIRT using finite differences on an image that is the non-linearly warped current test image (using the deformation field). Since the deformation field applies the same warp to all the diffusion images, we compute this gradient on each diffusion gradient, g*_j_*, as indicated in (11).

From (9) and (10),

(12) $$H\left(w\right)=\frac{2}{mn}\underset{j}{\sum }{\left(J^{j}\left(w\right)\right)}^{T}J^{j}\left(w\right)\in {\mathbb{R}}^{s\times s},$$

where **J***^j^*(**w**) is given by (11). Now, the gradient of the cost function is given by (see (7) and (9))

(13) $$\nabla C\left(w\right)\;=\;\frac{dC\left(w\right)}{dw}\;=\;\frac{2}{mn}\frac{dE^{T}}{dw}E\;=\;\frac{2}{mn}J^{T}\left(w\right)\left({\tilde{\text{I}}}_{test}\left(w\right)-I_{ref}\right)\in {\mathbb{R}}^{s}.$$

Until now, we have extended FNIRT to DW-MRIs and provided the equations to update **w**, from (8), (12), and (13), based on the deformation field model in FNIRT. However, this minimization algorithm still does not account for the intensity correction provided by angular interpolation. Hence, we need to extract the rotational component from the local affine matrix (see (4)),

(14) $$I+J_{u}\left(x_{i}\right)=R\left(x_{i}\right)S\left(x_{i}\right),i=1,\;\ldots ,\;n,$$

where **R**(**x***_i_*) and **S**(**x***_i_*) are respectively the rotational and strain components at each voxel. Hence, the transformed test image becomes

(15) $${\dot{\text{I}}}_{test}\left(w\right)={\left[{\dot{\text{I}}}_{test}^{T}\left(R^{T}\left(x_{1}\right)g,w\right)\ldots \;{\dot{\text{I}}}_{test}^{T}\left(R^{T}\left(x_{n}\right)g,w\right)\right]}^{T},$$

where, **g** = [**g**_1_ … **g***_m_*] and ${\dot{I}}_{test}\left({\text{R}}^{T}\left({\text{x}}_{i}\right)\text{g},\text{ w}\right)={\left[{\dot{I}}_{test}\left({\text{x}}_{i},\;{\text{R}}^{T}\left({\text{x}}_{i}\right){\text{g}}_{j}\text{w}\right)\right]}_{j}$ is the spatially and angularly interpolated test DW-MRI at **x***_i_*. Now, the Jacobian is given by

(16) $$J\left(w\right)={\left[\frac{\partial \left({\dot{\text{I}}}_{test}^{T}\left(R^{T}\left(x_{1}\right)g,w\right)\right)}{\partial w}\ldots \frac{\partial \left({\dot{\text{I}}}_{test}^{T}\left(R^{T}\left(x_{m}\right)g,w\right)\right)}{\partial w}\right]}^{T}.$$

As before, by the chain rule and (5),

(17) $$J^{j}(w)={\left[{\frac{\partial {\dot{I}}_{test}(x_{i},\;R^{T}(x_{i})g_{j},\;w)}{\partial x}|}_{{x^{\prime }}_{i}}B_{k}({x^{\prime }}_{i})\right]}_{jk}.$$

The Hessian can be computed as indicated in (12), and the gradient of the cost function as in (13), but with **J** as defined in (16), (17), and ${\dot{\text{I}}}_{test}\left(w\right)$ as defined in (15). It remains to define the local angular interpolation, given by,

(18) $${\dot{I}}_{test}\left(x_{i},R^{T}\left(x_{i}\right)g_{j},w\right)=\frac{\sum_{k=1}^{m}\alpha_{k}{Ĩ}_{test}\left(x_{i},g_{k},w\right)}{\sum_{k=1}^{m}\alpha_{k}},\;j=1,\ldots ,m.$$

As indicated in Equations (7), (12), and (13), the cost function, Hessian, and gradient are functions of the B-splines representing the displacement field. Hence, we can use the multi-scale Levenberg-Marquart (LM) minimization algorithm used in FNIRT (Andersson et al., 2007) to iteratively estimate the displacement field. At every iteration of the LM algorithm, the diffusion volumes are spatially registered using the local affine matrix (14) and their intensity interpolated using AI (15). This minimization method is commonly employed by other algorithms in FSL. The algorithm for non-linear registration using AI is detailed in **Algorithm 1**.

**Algorithm 1. Non-linear Registration with Angular Interpolation**



**Require:** Initial deformation field **u**_0_(**x**)
 (zero if not provided).
  **while** C(w) decreases do {OPTIMIZATION}
   **for** *i* = 1 → *n* **do**
    Compute the Jacobian and Hessian of
     the error **E** using (17) and (12).
    Compute the rotational component
      **R**(**x**_*i*_) of **I** + **J***_u_* (**x**_*i*_) as indicated
      in (14).
    Compute the overall cost *C*(**w**) as
      indicated in (7).
    Compute the gradient of the cost
      function ∇*C*(**w**) as indicated
      in (13).
    **w** ← **H**(**w**)^−1^∇ *C*(**w**).
    **for** *j* = 1 → *m* **do**
     Compute *Ĩ*_*test*_(**x**_*i*_, **g**_*j*_, **w**) using spatial 
      interpolation.
    **end for**
    Compute *İ*_*test*_ (**X***_i_*, **R***^T^* (**x***_i_*)**g***_j_*, **w**) using
      angular interpolation (15).
   **end for**
  **end while**



### 2.3 Experimental details

#### 2.3.1 Diffusion-weighted MRI acquisitions

Five diffusion-weighted MRI datasets were obtained on a 7T scanner (Magnex Scientific, UK) driven by a Siemens console (Erlangen, Germany), and using a Siemens head-gradient insert capable of 80 mT/m using a single refocused 2D single shot spin echo EPI sequence, 1.5 *mm* isotropic voxels, 128 directions at b = 1500 *s/mm*^2^ and 15 b0s (Lenglet et al., 2012). We also use two DW-MRI datasets obtained on a 3T scanner (Siemens MAGNETOM Trio) using whole-body gradients capable of 40 mT/m, a single refocused 2D single shot spin echo EPI sequence, 2 *mm* isotropic voxels, 128 directions at b = 1000 *s/mm*^2^ and 16 additional b0s (Zhan et al., 2013). Each DWI dataset was corrected for eddy current distortions and head motion using a 12 degree of freedom linear registration with the initial b0 image. They were also corrected for geometric distortions, by unwrapping the images using a field map. The diffusion gradients were reoriented to take into account the spatial transformations used for eddy current distortions correction [see details in Lenglet et al. (2012), Zhan et al. (2013)].

The nomenclature used here refers to these images as Subject1 to Subject6, the first four subjects correspond to the 7T data, while the last two correspond to the 3T data. Subject3 was scanned twice, at two different dates. We refer here to Subject3 and Subject3-2 to differentiate them. The research protocols used for the acquisition of the 7T and 3T data were approved by the Institutional Review Board (IRB) of the University of Minnesota. All subjects provided informed written consent prior to participating in the research protocols. We performed inter-subject registrations among all five datasets (subjects 1-4) acquired at 7T and separated inter-subject registrations between the pair of datasets acquired at 3T (Subjects 5 and 6).

There are 20 possible registrations between Subjects 1–4. Taking into account that Subject3 and Subject3-2 correspond to the same subject, we choose seven registration pairs (see Table 1), which cover all the intra-subject and inter-subject registration cases, without reversing the roles of the reference and test images, nor registering to the same subject twice. We also performed two registrations between Subjects 5 and 6 as indicated in Table 1.

**Table 1:** **Tested registration pairs**.

Test image	Reference image						
	Subject1	Subject2	Subject3	Subject4	Subject3-2	Subject5	Subject6
Subject1		X	X	X			
Subject2			X	X			
Subject3				X	X		
Subject5							X
Subject6						X	

Linear registrations in FSL are performed with FLIRT and the new FLIRT-based AI using cross-correlation as the distance measure between images (see Section 2.1).

#### 2.3.2 Synthetic transformations

In order to obtain unbiased synthetic transformations, we use the advanced normalization tools (ANTs)^12^ to warp spatially a DW-MRI dataset Subject2 using a pre-defined random set of affine and non-linear transformations. ANTs uses one of the top five MRI non-linear registration tools identified in a recent evaluation study Klein et al. (2009). We generated 20 random affine matrices (see Kannala et al., 2005, for instance) of the form **A** = **RSD**, where **R** is a pure rotation matrix, **S** an upper triangular skew matrix, and **D** a diagonal matrix providing scale change (see details in Section 1.1 of the Appendix). We also generated 20 random non-linear deformation fields, based on the work of Noblet et al. (2006) (see details in Section 1.2 of the Appendix). These non-linear deformation fields ensures a topology preserving transformation (Noblet et al., 2006). As indicated in the introduction, registration of DW-MRIs requires also intensity corrections to reflect the change in fiber orientation. To avoid bias in favor of our proposed technique, we do not use here our AI method to perform the intensity correction [Equation (18)]. Instead, we use an eighth order spherical harmonics (SH) representation of the DW-MRI datasets. Using this SH basis, it is straightforward to interpolate the intensities at different gradient directions (see Dhollander et al. (2010); Bouix et al. (2010); Kamath et al. (2012), for instance). We must point out here that while the SH representation of DW-MRIs can smooth out certain features of the diffusion signal (at each particular *b*-value), it provides at least an unbiased correction of the image intensities that will be used for all the other algorithms tested. In addition, we only use ANTs to perform the spatial warping of each diffusion direction with a known pre-defined transformation. We do not use ANTs to try to register the warped images, since that would bias the results toward ANTs, as it was used to perform the warp in the first place.

**Figure 1:**
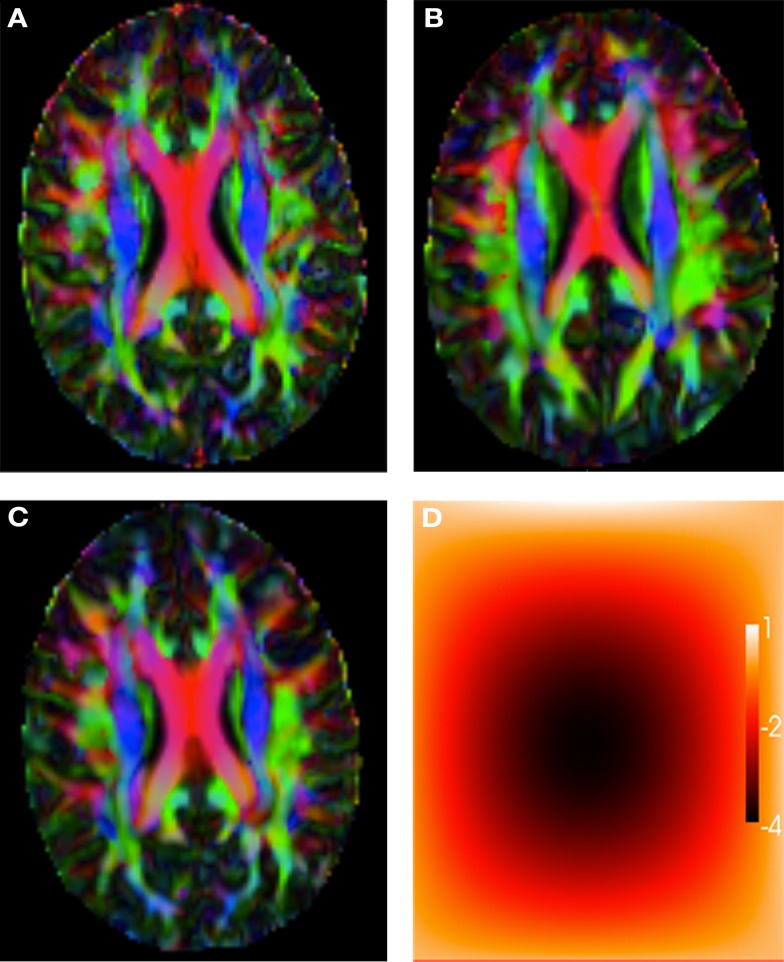
**(A)** Reference image. **(B)** Affinely warped reference image. **(C)** Non-linearly warped reference image using a small displacement field, **(D)** Non-linear small deformation field.

Figure 1A shows the main direction of diffusion in the reference image, color coded with an RGB colormap (red for left-right, green for antero-posterior, and blue for superior-inferior), with brightness modulated by the value of the FA. Figure 1B shows the main direction of diffusion (same color code as before) for a linearly transformed test image. Figure 1C shows the main direction of diffusion for a non-linearly warped test image, and Figure 1D shows the corresponding non-linear deformation field, color coded by the magnitude of the displacement.

#### 2.3.3 Intra- and inter-subject registration

In the previous section, we used known linear and non-linear transformations, since it allows us to consider the reference image as ground truth and therefore evaluate the registration accuracy. On the other hand, there is no consensus on how to evaluate the registration accuracy for any pair of images (Kannala et al., 2005; Noblet et al., 2006). In fact, the optimal registration of a pair of images depends largely on the application and registering two different subjects should not reduce or eliminate the structural differences between different brains. Nevertheless, and for completeness, we also tested the registration algorithms for pairs of different DW-MRIs, even though registration errors and anatomical variability cannot be differentiated from each other in this case.

Non-linear registration between any pair of DW-MR images requires to conduct first an affine registration. We will use here the best affine registration found, so that all the non-linear registration algorithms start with the same linearly registered image.

#### 2.3.4 Evaluation of results

We compare the results from linear and non-linear registration using b0-based, DTI-based, and DWI-based registration. The registration error in terms of the mean squared error (*MSE*) is given by

(19) $$MSE=\frac{1}{mn}\overset{n,m}{\underset{i=1,j=1}{\sum }}{\left|\right.\left|\right.\frac{I_{test}\left(x_{i},g_{j}\right)-I_{ref}\left(x_{i},g_{j}\right)}{I_{max}}\left|\right.\left|\right.}_{2}^{2}\times 100,$$

where *n* is the total number of voxels, *m* the number of gradient directions in the reference image, and we are normalizing the error by the maximum intensity in the reference image *I_max_*. Based on Wang et al. (2011), we also define the mean fiber orientation error as

(20) $$foe=\frac{1}{n}\overset{n}{\underset{i=1}{\sum }}cos^{-1}|\left\langle v_{i}^{test},v_{i}^{ref}\right\rangle |,$$

where ${\text{v}}_{i}^{ref}$, ${\text{v}}_{i}^{test}$ are respectively, the estimated fiber orientations of the reference and registered DW-MR images at the *i*-th voxel.

The fiber orientations of the linearly and non-linearly registered images correspond to the first eigenvector of the second order tensor model for the reference and registered test images. The second order tensors (DTI) required by DTI-TK were computed using DTIFIT from FSL. MedINRIA on the other hand, it uses its own algorithm to estimate the tensors from the diffusion volumes and diffusion gradients/*b*-value(s) information, so we use those within MedINRIA. DTI-TK and MedINRIA can register scalar and tensor volumes. Hence, the first eigenvector can be extracted from the registered tensors using DTI-TK interoperability tools, which also works for MedINRIA, since they are both NIFTI^13^ compliant. Given that FLIRT and FNIRT can only register scalar volumes, we generate registered DW-MRIs by applying the transformation found (using the b0 volume) by FLIRT and FNIRT to each one of the volumes in the DW-MR data set. Intensity correction was also applied to these spatially registered datasets using SH. From these registered DW-MRIs, we can compute the second order tensors using DTIFIT. Similarly, we can use the spatial transformation (affine matrix or deformation field) found by DTI-TK and MedINRIA to spatially register every volume in the DW-MR datasets, applying optionally SH intensity correction. This allows us to obtain registered DW-MR datasets for FLIRT, FNIRT, DTI-TK, and MedINRIA. The advantage of obtaining registered DW-MRIs for all methods considered here is that we can then compute, in an unbiased fashion, the registration *MSE* from the registered DW-MR datasets with and without intensity correction. It also allows us to compare the registration *foe* of these registered DW-MRIs with the *foe* of the registered DTIs and the proposed FLIRT-based and FNIRT-based AI registration algorithms.

The second order tensors (DTI) can be computed from the linearly and spatially only registered DW-MRIs (i.e., without SH correction), by providing the reoriented gradients ${{\text{g}}^{\prime }}_{j}={\text{R}}^{T}{\text{g}}_{j},j=1,\;\ldots ,\;m$, to DTIFIT (see Section 2.1), where **R** is the rotational component of the affine matrix used to spatially register the DW-MRIs.

The registration *foe* was computed on forty^14^ regions of interest (ROIs) taken from the John Hopkins University (JHU) white matter atlas (Hua et al., 2008)^15^. The JHU white matter regions were registered (using the FA volumes) from the atlas to each one of the DW-MR reference datasets. The *MSE* was also computed on the the same brain regions.

## 3 Results

### 3.1 Linear registration

Table 2 summarizes the results of all the linear registration experiments, as described in Section 2.3, in terms of the mean *foe* and *MSE*, with their corresponding standard deviation, within parenthesis. Since only two 3T datasets were used to illustrate the performance of the algorithms at lower spatial resolution, we do not report here the standard deviation of the registration error. FLIRT4D-AI indicates the proposed registration of (4D) DW-MR datasets using angular interpolation. The appended suffix – *tensors* indicates that the *foe*s were estimated directly from the tensors registered using the corresponding registration method. The appended suffix – *volumes* indicates that the affine transformations found by the corresponding registration method were used to spatially register all the diffusion volumes of the test DW-MRIs. Finally, the appended suffix – *SH* indicates that SH intensity correction was performed on top of the spatial registration. There were 20 affine registrations performed, for all experiments performed using random synthetic affine warps (see Section 2.3.2) and 9 intra- and inter-subject affine registrations, seven of which are indicated in Table 1, and the remaining two correspond to registering back and forth the two 3T datasets (see Section 2.3.3).

**Table 2:** **Linear registration (best results are indicated in bold)**.

Method	Registration of 20 synthetic affinely warped DW-MRIs		Intra- and Inter-subject registration of DW-MRIs			
			7T		3T	
	*foe*	*MSE*	*foe*	*MSE*	*foe*	*MSE*
FLIRT4D-AI	14.5 (1.2)	1.43 (0.3) × 10^−3^	27.5 (3.4)	1.11 (0.2) × 10^−3^	22.6	0.99 × 10^−3^
FLIRT-volumes	19.2 (3.3)	2.43 (0.7) × 10^−3^	26.7 (3.1)	1.11 (0.2) × 10^−3^	22.5	0.99 × 10^−3^
FLIRT-volumes-SH	19.3 (3.3)	2.45 (0.7) × 10^−3^	26.8 (3.1)	1.06 (0.2) × 10^−3^	22.5	0.92 × 10^−3^
DTI-TK-tensors	16.0 (1.4)	–	**24.0 (2.3)**	–	**21.1**	–
DTI-TK-volumes	16.3 (1.4)	**1.23 (0.3)** × 10^−3^	24.7 (2.5)	1.07 (0.2) × 10^−3^	23.6	0.86 × 10^−3^
DTI-TK-volumes-SH	15.7 (1.4)	1.26 (0.3) × 10^−3^	26.0 (2.0)	1.06 (0.2) × 10^−3^	21.8	**0.80** × 10^−3^
MedINRIA-tensors	13.8 (0.7)	–	24.0 (2.5)	–	22.0	–
MedINRIA-volumes	9.6 (0.3)	1.35 (0.3) × 10^−3^	24.5 (2.4)	1.09 (0.2) × 10^−3^	28.6	0.92 × 10^−3^
MedINRIA-volumes-SH	**8.5 (0.4)**	1.26 (0.3) × 10^−3^	24.2 (2.3)	**1.05 (0.2)** × 10^−3^	21.6	0.86 × 10^−3^

Conversion from the ITK^16^ matrix format used by MedINRIA to the standard 4 × 4 affine matrix was done using the *c3d_affine_tool*^17^. DTI-TK matrix format directly provides the 3 × 3 submatrix of the affine transformation plus the displacement. Hence, the rotational component of the affine matrix can be easily extracted in DTI-TK. The rotational component of the affine registration matrices found by all the algorithms was computed using the *decompose_aff* method in FSL. We used the *baloo* (Ourselin et al., 2000), which is the affine registration algorithm in MedINRIA 2.01^18^ and worked very well in all cases considered.

MedINRIA achieved the lowest *foe* as well as very low *MSE* using the affine transformation to spatially register each diffusion volume and then performing SH intensity correction. The *MSE* correlates for the most part with the *foe*, but not always. For instance, DTI-TK-volumes have the lowest *MSE* in Table 2, for the synthetic affinely warped datasets, but it also has the largest *foe*. This could indicate that minimizing the *MSE* does not necessarily reduces the *foe*. Also, The SH intensity correction helps reduce the *foe* in some, but not in all cases. This can be understood from the fact that if the spatial registration was not accurate, then the SH correction is also going to be inaccurate and could even increase the error, as can be appreciated in the FLIRT-volumes-SH results compared to FLIRT-volumes. Notice also that the *foe* is low in MedINRIA-volumes despite not having intensity correction using SH. This is due to the fact that we are providing the reoriented diffusion gradients, nonetheless, the *foe*s and *MSE* are even lower using the SH intensity correction, which confirms the importance of adjusting the intensity of spatially registered diffusion volumes.

A paired t-test reveals that there is significant statistical difference in favor of SH intensity correction, which reduced the *foe* by almost 12% (p = 0.0015), in the synthetic transformations. FLIRT4D-AI has a much lower *foe* and *MSE* than FLIRT-volumes as well as a lower variance. However, no significant statistical difference was found in this case, given the large variance of FLIRT-volumes results. Overall, the results indicate that performing intensity correction is important, but the accuracy of the registration algorithm is obviously critical, as can be seen from the very good results of MedINRIA. The fact that MedINRIA did better than the other methods, including FLIRT4D-AI, could be attributed to the fact that its affine registration algorithm is superior to the other registration methods considered here.

**Figure 2:**
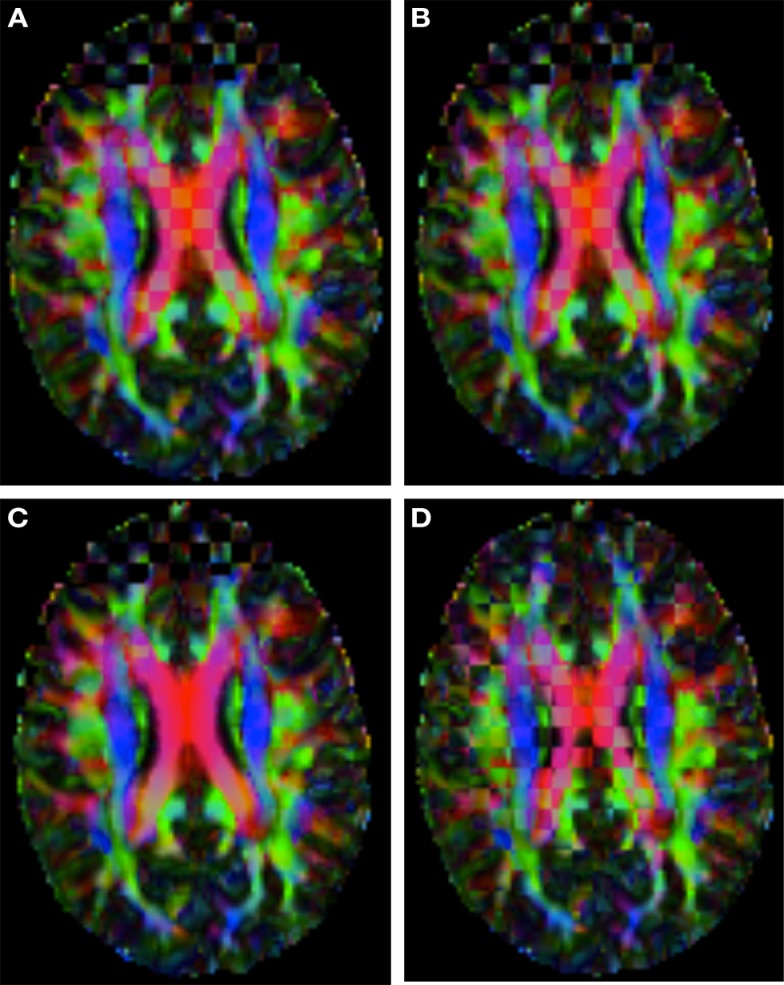
**Checkerboard comparison of the RGB color coded main diffusion direction between Subject2 and a registered random affine transformation of Subject2 using (A) FLIRT-volumes-SH**. **(B)** FLIRT4D-AI. **(C)** MedINRIA-volumes-SH, **(D)** DTI-TK-volumes-SH.

**Figure 3:**
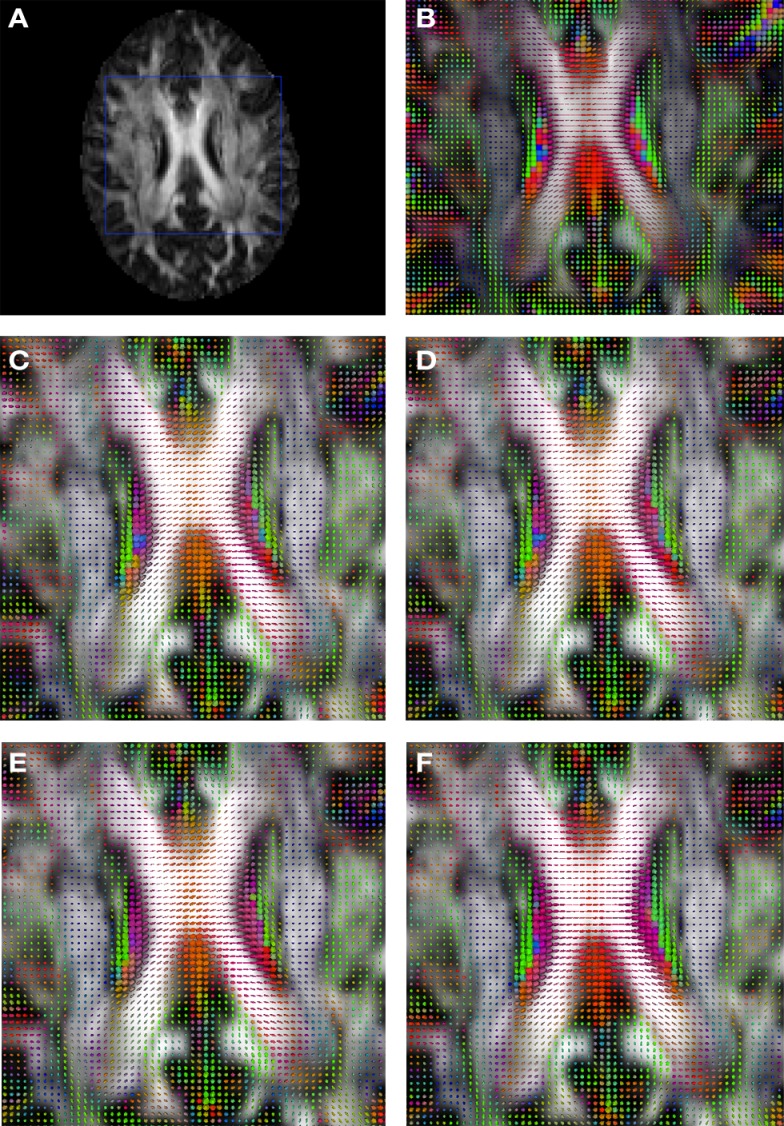
**Comparison of the tensors (represented as RGB color coded glyphs) of Subject2 and the registered tensors of a random affine transformation of Subject2**. **(A)** Subject2 FA indicating the region selected to show the tensors. **(B)** Subject2 tensors. Registered tensors using **(C)** FLIRT-volumes-SH, **(D)** FLIRT4D-AI, **(E)** MedINRIA-volumes-SH, and **(F)** DTI-TK-volumes-SH.

**Figure 4:**
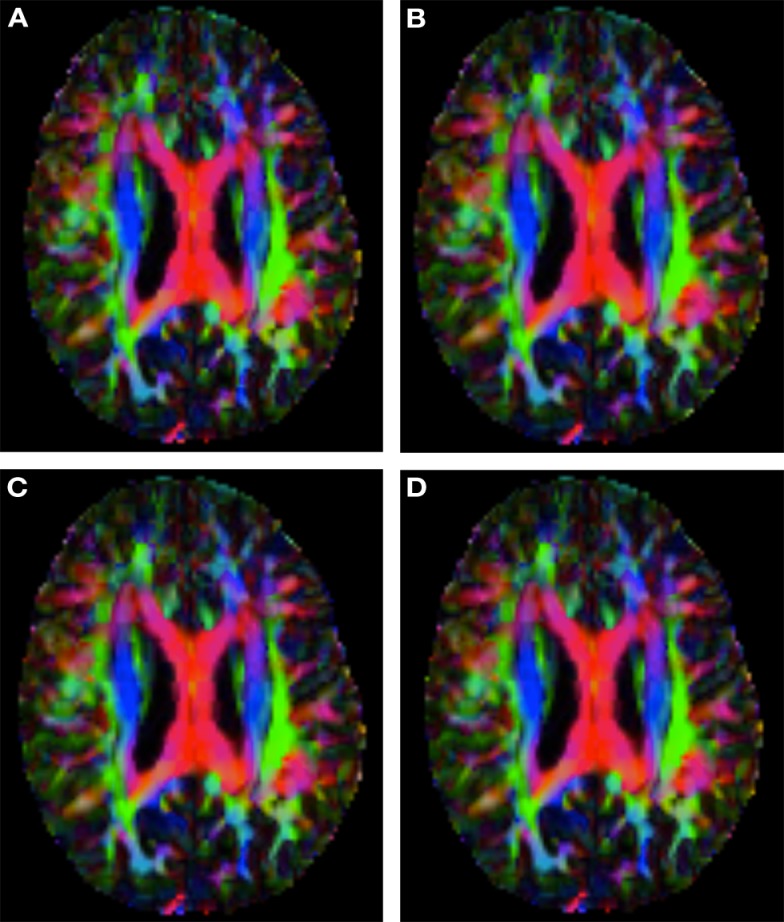
**Checkerboard comparison of the RGB color coded main diffusion direction between Subject3-2 and Subject3 registered to Subject3-2 using (A) FLIRT-volumes-SH**. **(B)** FLIRT4D-AI. **(C)** MedINRIA-volumes-SH, **(D)** DTI-TK-volumes-SH.

**Figure 5:**
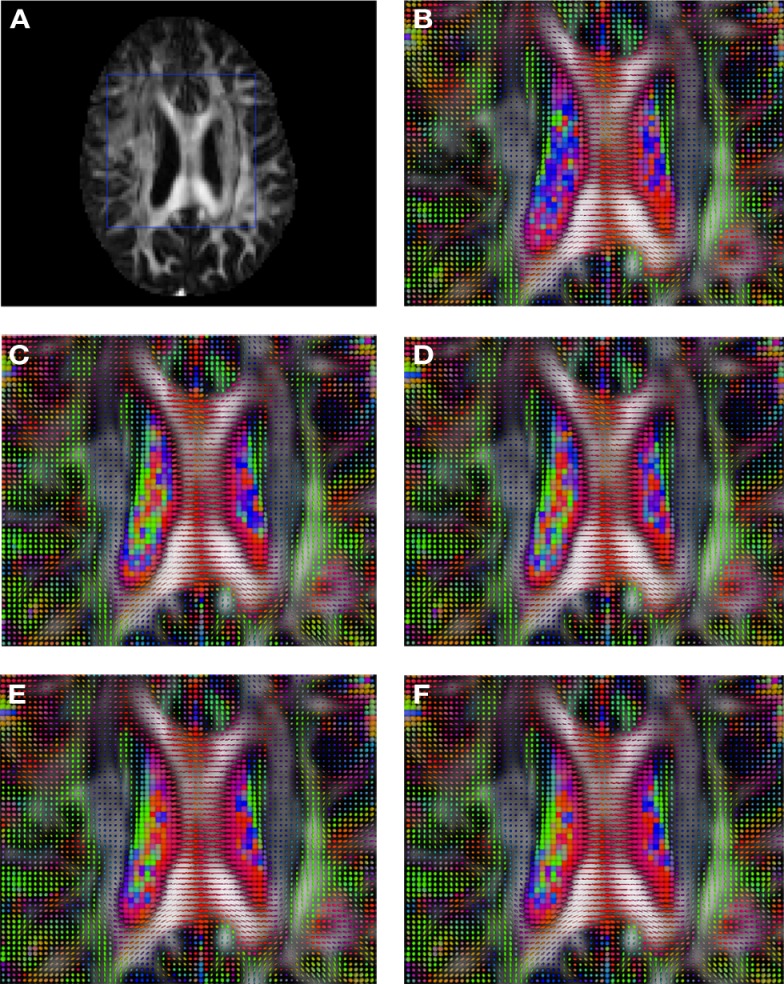
**Comparison of the tensors (represented as RGB color coded glyphs) of Subject3-2 and the tensors Subject3 registered to Subject3-2**. **(A)** Subject3-2 FA indicating the region selected to show the tensors. **(B)** Subject3-2 tensors. Registered tensors of Subject3 using **(C)** FLIRT-volumes-SH, **(D)** FLIRT4D-AI, **(E)** MedINRIA-volumes-SH, and **(F)** DTI-TK-volumes-SH.

Figure 2 shows a checkerboard comparison between the main diffusion direction (RGB color coded) of the reference DW-MRI (Subject2) and one of the synthetic affine transformations. Figure 3 shows the tensors (represented as RGB color coded glyphs) of the reference (Subject2) and the registered images indicated in Figure 2. The best performance of MedINRIA can be appreciated in these figures, where the checkerboard effect is lower than in the other methods, followed by DTI-TK, FLIRT4D-AI, and finally FLIRT-volumes. Figure 4 shows a checkerboard comparison between the main diffusion direction in Subject3-2 and Subject3 registered to Subject3-2. Figure 5 shows the tensors (represented as RGB color coded glyphs) of the reference (Subject3-2) and Subject 3 registered to Subject3-2. Since this is the same individual, the registration errors are much lower than between different subjects. In terms of the mean *foe* FLIRT4D-AI has the lowest error (10.1), followed by FLIRT4D (10.6), DTI-TK (11.0), and MedINRIA (11.7).

### 3.2 Non-linear registration

Table 3 summarizes the results of all the non-linear registrations indicated in Section 2.3, in terms of the mean *foe* and *MSE*, with their corresponding standard deviation, except for the two 3T datasets, where only the mean is reported.

**Table 3:** **Non-linear registration (best results are indicated in bold)**.

Method	Registration of 20 synthetic affinely warped DW-MRIs		Intra- and Inter-subject registration of DW-MRIs			
			7T		3T	
	*foe*	*MSE*	*foe*	*MSE*	*foe*	*MSE*
FLIRT4D-AI	**3.6 (0.1)**	1.0 (0.01) × 10^−4^	19.8 (1.9)	2.9 (0.3) × 10^−4^	18.4	**6.1** × 10^−4^
FLIRT-volumes	3.8 (0.1)	1.4 (0.01) × 10^−4^	21.5 (1.8)	3.2 (0.4) × 10^−4^	20.9	6.6 × 10^−4^
FLIRT-volumes-SH	4.2 (0.2)	1.1 (0.02) × 10^−4^	21.5 (1.8)	3.1 (0.4) × 10^−4^	20.8	6.3 × 10^−4^
DTI-TK-tensors	4.5 (0.2)	–	**13.4 (0.7)**	–	**13.3**	–
DTI-TK-volumes	4.3 (0.2)	1.1 (0.03) × 10^−4^	15.1 (1.0)	3.3 (0.5) × 10^−4^	24.0	9.3 × 10^−4^
DTI-TK-volumes-SH	4.2 (0.2)	1.2 (0.03) × 10^−4^	16.5 (1.2)	3.3 (0.5) × 10^−4^	27.9	10.0 × 10^−4^
MedINRIA-tensors	5.7 (0.4)	–	29.3 (2.0)	–	23.0	–
MedINRIA-volumes	4.7 (0.3)	**0.6 (0.07)** × 10^−4^	25.9 (1.9)	**2.6 (0.3)** × 10^−4^	27.1	8.1 × 10^−4^
MedINRIA-volumes-SH	6.5 (0.5)	1.4 (0.07) × 10^−4^	32.6 (2.2)	2.7 (0.3) × 10^−4^	30.6	8.7 × 10^−4^

The notation is the same as in Table 2, except that now FLIRT is replaced by FNIRT. Also, the non-linear registration used in MedINRIA corresponds to the diffeomorphic demons algorithm. In this case the appended suffix −*volumes* indicates that the corresponding registration algorithm used the estimated deformation field to non-linearly register each one of the diffusion volumes. The appended suffix −*SH* indicates that besides non-linear spatial registration, the intensity of the diffusion volumes is corrected using SH q-space interpolation and the rotational component of the equivalent local affine transformation [see Equation (14)]. The local affine transformation depends on the Jacobian of the deformation field [Equation (14)], which was computed using the *deffield2jacobian* method in FSL. The estimated deformation fields in MedINRIA and DTI-TK were converted to FSL format using the NIFTI Matlab toolbox^19^.

Table 3 indicates that the lowest *foe* for the synthetic non-linearly warped DW-MRIs correspond to FNIRT4D-AI, while the best *foe* for the intra and inter-subject registration was achieved by DTI-TK, which coincides with previous studies reporting that DTI-TK performed very well when compared to other tensor-based non-linear registration algorithms (Wang et al., 2011). MedINRIA (demons registration algorithm) did not perform as well as FNIRT or DTI-TK. Our results also indicate that SH correction did not improve any of the results obtained using non-linear spatial registration. This might be due to the rather coarse internal representation of deformation fields in FSL that is based on splines (as used in FNIRT). A paired t-test indicates that FNIRT4D-AI has a 6% lower error than FNIRT in the synthetic transformations (p = 0.001). The fact that DTI-TK performed better than FNIRT4D-AI for intra- and inter-subject registration possibly indicates that differences between the underlying algorithms are bigger than the gain obtained using AI or SH. This also corroborates our previous results on linear registration, where MedINRIA performed optimally. This is also inevitable for such a study, where various algorithms, with various strengths, and weaknesses are compared.

**Figure 6:**
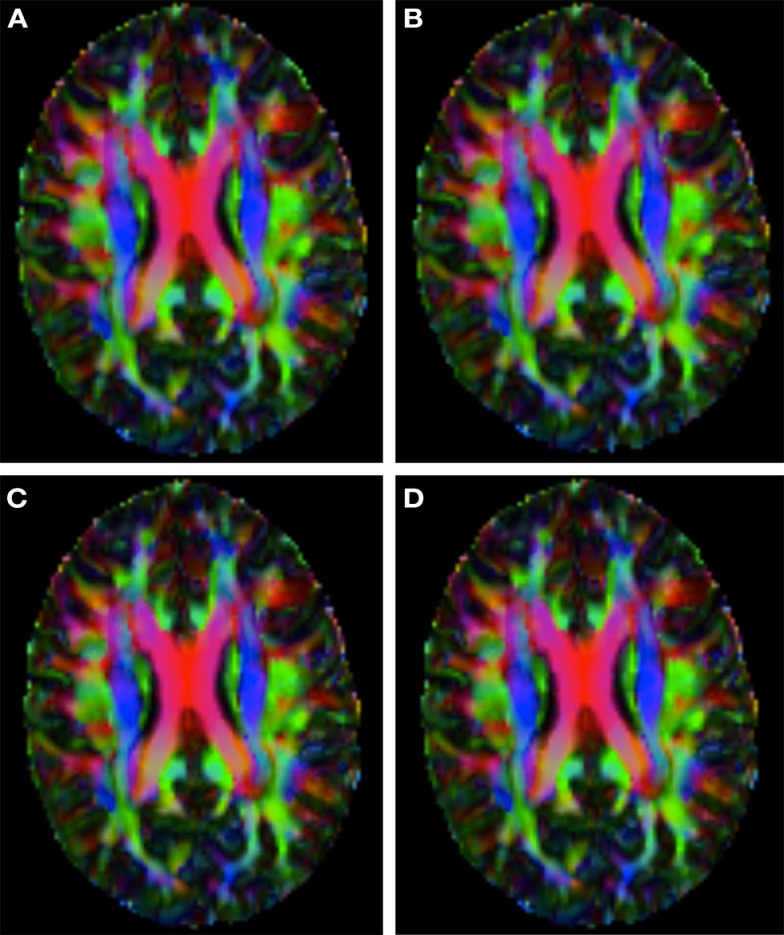
**Checkerboard comparison of the RGB color coded main diffusion direction between Subject2 and a registered random non-linear transformation of Subject2 using (A) FNIRT-volumes-SH**. **(B)** FNIRT4D-AI. **(C)** MedINRIA-volumes-SH, **(D)** DTI-TK-volumes-SH.

**Figure 7:**
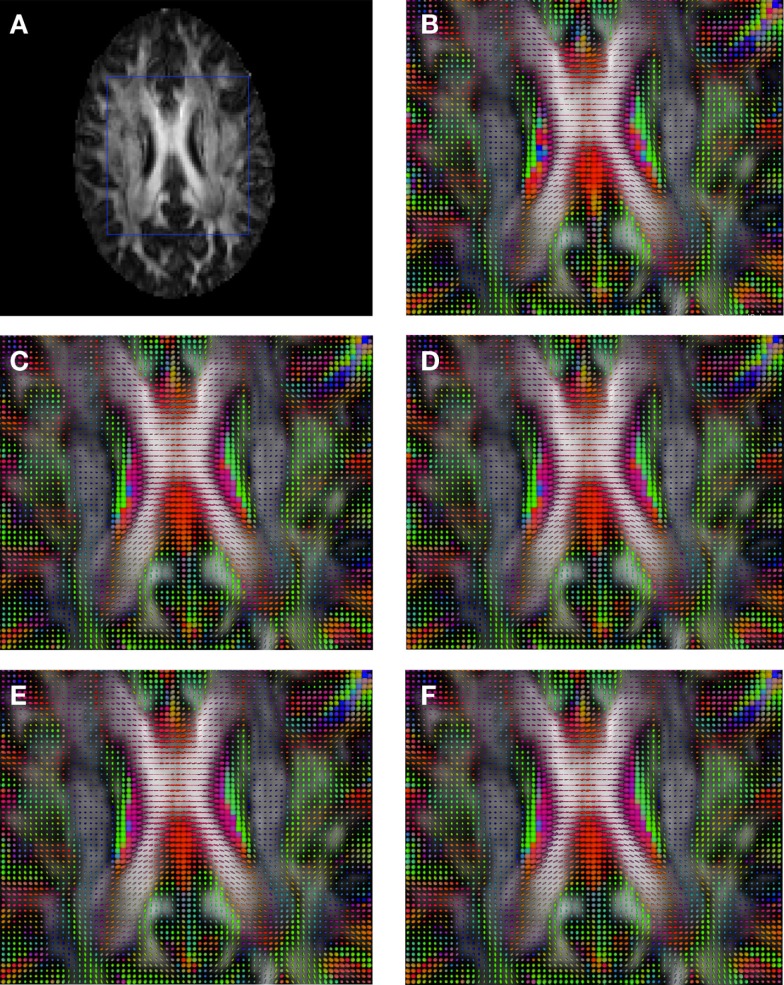
**Comparison of the tensors (represented as RGB color coded glyphs) of Subject2 and the tensors of a registered random non-linear transformation of Subject2**. **(A)** Subject2 FA indicating the region selected to show the tensors. **(B)** Subject2 tensors. Registered tensors using **(C)** FLIRT-volumes-SH, **(D)** FLIRT4D-AI, **(E)** MedINRIA-volumes-SH, and **(F)** DTI-TK-volumes-SH.

**Figure 8:**
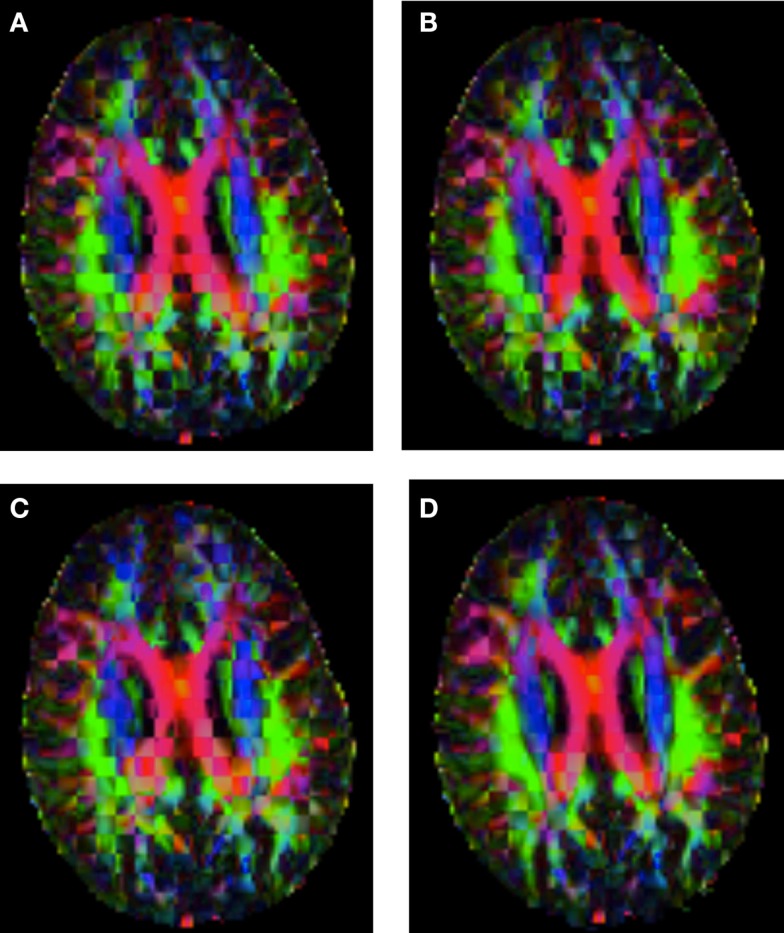
**Checkerboard comparison of the RGB color coded main diffusion direction between Subject3 and Subject2 registered to Subject3 using (A) FNIRT-volumes-SH**. **(B)** FNIRT4D-AI. **(C)** MedINRIA-volumes-SH, **(D)** DTI-TK-volumes-SH.

**Figure 9:**
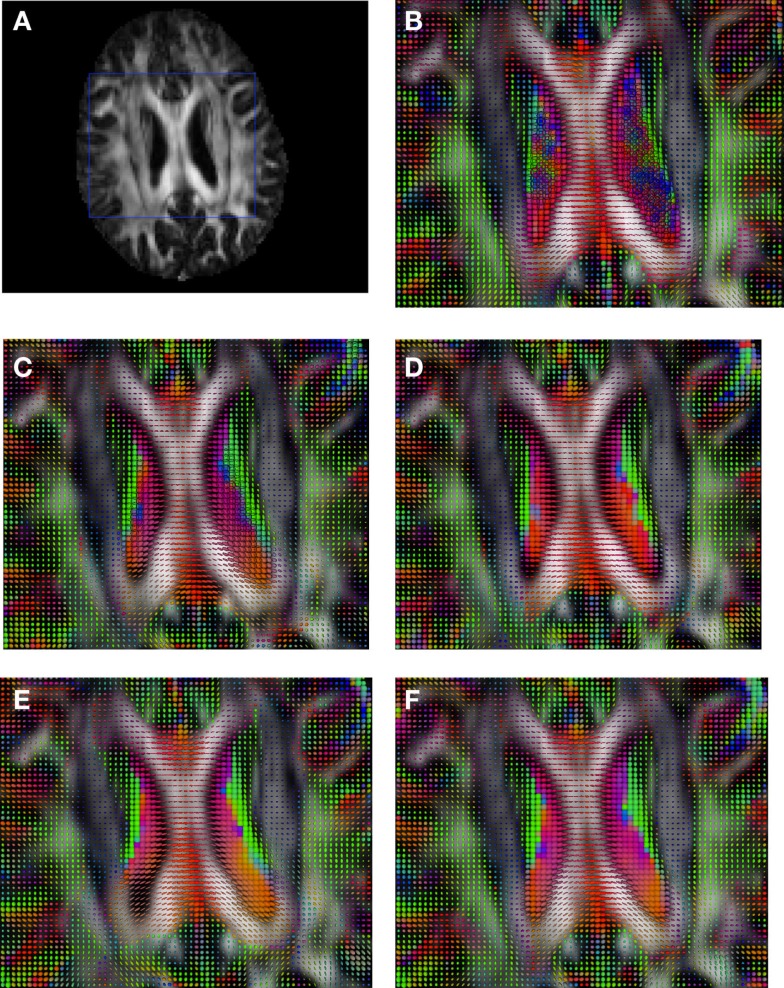
**Comparison of the tensors (represented as RGB color coded glyphs) of Subject3 and the tensors of Subject2 registered to Subject3**. **(A)** Subject3 FA indicating the region selected to show the tensors. **(B)** Subject3 tensors. Registered tensors of Subject2 using **(C)** FLIRT-volumes-SH, **(D)** FLIRT4D-AI, **(E)** MedINRIA-volumes-SH, and **(F)** DTI-TK-volumes-SH.

Figure 6 shows a checkerboard comparison between the main diffusion direction (RGB color coded) of the reference DW-MRI (Subject2) and one of the registered synthetic random non-linear warped DW-MRIs. Figure 7 shows the tensors (represented as RGB color coded glyphs) of the reference (Subject2) and the registered images indicated in Figure 6. The best performance of FNIRT4D-AI and FNIRT-volumes is clear over the other methods. Figure 8 shows a checkerboard comparison between the main diffusion direction in Subject3 and Subject2 registered to this subject. Figure 9 shows the tensors (represented as RGB color coded glyphs) of the reference (Subject3) and Subject2 registered to Subject3. As expected, there are many differences between the reference and registered image, since they correspond to different individuals. However, it is not difficult to see that the registered images using DTI-TK followed by FNIRT4D-AI have lower differences with respect to the reference, compared to FNIRT-volumes and MedINRIA.

## 4 Discussion

Previous work using higher order models (Barmpoutis et al., 2007; Cheng et al., 2009; Dhollander et al., 2010; Verma and Bloy, 2010; Yap et al., 2010; Du et al., 2011; Geng et al., 2011) compared their proposed model with DTI or single volume registration, using exactly the same underlying registration algorithm, where the difference is due only to the model used. We addressed here a slightly more challenging task using different registration algorithms (with the exception of FLIRT and FNIRT in FSL), in order to provide a useful and more practical comparison. The intrinsic differences among the different registration algorithms analyzed here can be larger than the differences found using AI or SH intensity correction. Consequently, AI should also be implemented within other registration algorithms, in order to make it competitive with other well-known and tested state of the art registration algorithms (based on second order tensors or higher order models such as the ODF), a representative set of which has been used in this study. Nonetheless, we have clearly demonstrated that AI improves the registration accuracy in many cases over existing state-of-the-art algorithms. We also provided a framework and software to perform FNIRT-based AI registration or just intensity correction, which will enable future comparative studies between various registration algorithms using different diffusion models.

Despite the strong differences between the different algorithms used here, the results indicate that registration of DW-MR datasets should include intensity correction within the minimization algorithm, as proposed in our extension of FNIRT. Intensity correction as a post-processing step after spatial correction does not necessarily improve results, and in fact can increase the *foe*. In addition, minimizing the *MSE* does not necessarily minimize the *foe*. We proposed here to use angular interpolation of q-space rather than SH interpolation, since it is simpler and uses the information in the image to perform intensity correction in the DW-MRIs, while SH uses a smooth basis that might lead to loss of information. Hence, AI should provide a better intensity correction than SH. Since AI is currently based on the intensity of the images, future work should address the possibility of extending the idea of AI to use a different feature.

Finally, no significant difference was found between the registration errors using the 3T dataset and the 7T datasets. This indicates that the algorithms studied can be applied to clinical data, where 3T datasets are more common than 7T data.

## Conflict of Interest Statement

The authors declare that the research was conducted in the absence of any commercial or financial relationships that could be construed as a potential conflict of interest.

## Figures and Tables

**Figure A1:**
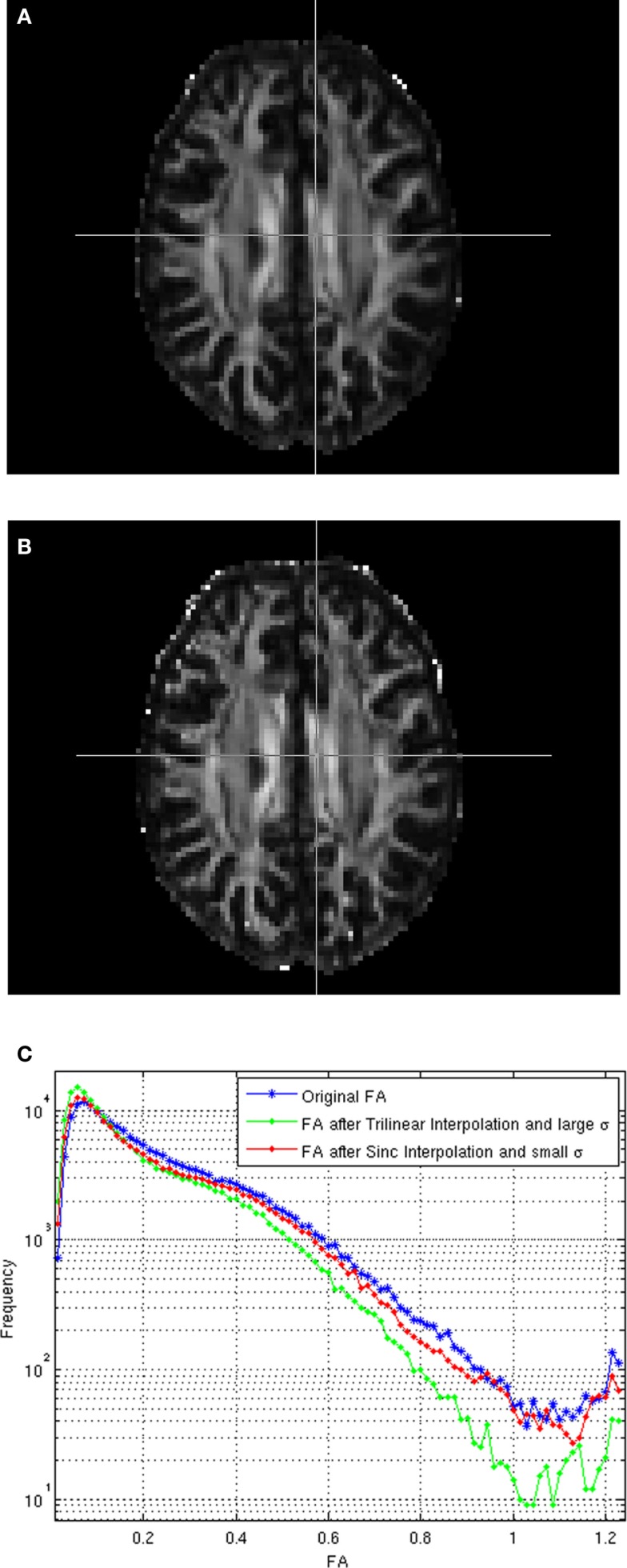
**(A)** FA of the registered image using trilinear interpolation and large σ. **(B)** FA registered using *sinc* interpolation and small σ, **(C)** Histograms of the FAs.

## Footnotes

^1^FMRIB’s linear image registration tool (http://fsl.fmrib.ox.ac.uk/fsl/flirt/).

^2^FMRIB’s non-linear image registration tool (http://www.fmrib.ox.ac.uk/fsl/fnirt/index.html).

^3^http://www.fmrib.ox.ac.uk/fsl.

^4^http://www.nitrc.org/projects/dtitk.

^5^http://www-sop.inria.fr/asclepios/software/MedINRIA.

^6^The most recent version of DTI-TK uses the PPD reorientation algorithm.

^7^Currently available at www.nitrc.org/projects/dwiregistration/.

^8^https://www.fmrib.ox.ac.uk/phpwiki/index.php/FSL%20Plugins.

^9^Usually nearest neighbor, trilinear, spline, or windowed sinc interpolation.

^10^FNIRT accepts 4D images, but works only with the first volume it encounters and disregards the remaining volumes.

^11^FNIRT minimization algorithm is a gradient descent approach, hence, its extension to other non analytically derivable cost functions is not straightforward.

^12^http://www.picsl.upenn.edu/ANTS/

^13^http://nifti.nimh.nih.gov/nifti-1.

^14^We discarded 8 regions (see supplementary material) from the 48 white matter labels in the JHU atlas, located in the lower brain areas and that were not always fully visible in our datasets.

^15^Available within the FSL library.

^16^http://www.itk.org/

^17^http://www.itksnap.org/pmwiki/pmwiki.php?n=Convert3D.Documentation

^18^http://med.inria.fr/

^19^http://www.mathworks.com/matlabcentral/fileexchange/8797.

## Appendix

### Generation of synthetic deformations

The following matrices **R**, **S**, and **D** provide random three-dimensional rotations, skewness, and change of scale, respectively

$$\begin{array}{llll} R & =\left[\;\begin{array}{cccc} 1 & 0 & 0 & 0\\ 0 & cos\alpha & -sin\alpha & 0\\ 0 & sin\alpha & cos\alpha & 0\\ 0 & 0 & 0 & 1 \end{array}\right]\left[\;\begin{array}{cccc} cos\beta & 0 & sin\beta & 0\\ 0 & 1 & 0 & 0\\ -sin\beta & 0 & cos\beta & 0\\ 0 & 0 & 0 & 1 \end{array}\right] & & \\ & \;\times \left[\;\begin{array}{cccc} cos\gamma & -sin\gamma & 0 & 0\\ sin\gamma & cos\gamma & 0 & 0\\ 0 & 0 & 1 & 0\\ 0 & 0 & 0 & 1 \end{array}\right], & & \\ S & =\left[\;\begin{array}{cccc} 1 & a & b & 0\\ 0 & 1 & c & 0\\ 0 & 0 & 1 & 0\\ 0 & 0 & 0 & 1 \end{array}\right],\;D=\left[\;\begin{array}{cccc} \delta & 0 & 0 & 0\\ 0 & \delta & 0 & 0\\ 0 & 0 & \delta & 0\\ 0 & 0 & 0 & 1 \end{array}\right], & & \end{array}$$

where *α*, *β*, *γ* are uniform random rotation angles along the *x*, *y*, and *z* axis respectively in the [−15°, 15°] range; *a*, *b*, *c* are uniform random numbers in the −0.125, 0.125] range; and *δ* is a scale uniform random number in the [0.875, 1.125] range.

#### Random nonlinear warps

The following equation defines the displacement field **u**(**x**) along the *x*, *y*, and *z* axis,

$$u\left(x\right)=\left\{\begin{array}{c} a_{x}sin\left(\frac{w\pi x}{n_{x}}\right)\;\\ a_{y}sin\left(\frac{w\pi y}{n_{y}}\right)\;\\ a_{z}sin\left(\frac{w\pi z}{n_{z}}\right)\;\\ \; \end{array}\right.\;s.t.\left(\frac{\left|a_{x}\right|}{n_{x}}+\frac{\left|a_{y}\right|}{n_{y}}+\frac{\left|a_{y}\right|}{n_{y}}\right)\leq \frac{1}{w\pi },$$

where *n_x_*, *n_y_*, *n_z_* are the number of voxels along the *x*, *y*, *z* axis, respectively, and *w*, *a_x_*, *a_y_*, *a_z_* are uniform random variables satisfying the indicated conditions.

### Implementation details

#### Effect of Interpolation on FA

The effect of spatial interpolation on DT-MRIs has been recently studied (Chao et al., 2009), indicating that the measured Fractional Anisotropy (FA) can be significantly reduced if linear interpolation is used. Basically, linear interpolation acts as a low-pass filter with equal weights that averages the tensors, leading to tensor swelling (Arsigny et al., 2005) and a reduction of the estimated FA values. We have not found an equivalent study for the effect of spatial interpolation of DW-MRIs on the estimated FAs, but we were able to see the same effect on the estimated FA values from the registered DW-MRIs when tri-linear interpolation is used, which is the default interpolation in FSL. In addition, we also found that using the recommended values in Tao and Miller (2006) for the standard deviation in (3) and (19), AI can also reduce the calculated FA values. Notice that AI can also be seen as a Gaussian filter, with zero mean and standard deviation σ defining the weights of the interpolating window.

We address this problem by using a Blackman windowed *sinc* interpolation filter (provided by FSL) for the spatial interpolation and reducing the standard deviation of the Gaussian coefficients for AI, in such a way that only the volumes closest to the specified gradient are accounted for. Only the closest gradients in the image should indeed be used to interpolate along a given gradient direction, otherwise, volumes with different gradients (and very different intensity responses) could affect the intensities computed for a new gradient direction.

Figure A1A shows the estimated FA for the registered test image using trilinear interpolation and the smoothing parameter σ computed from the average of the geodesic distances between neighboring gradients, as indicated in Tao and Miller (2006), Figure A1B shows the estimated FA for the registered test image using windowed *sinc* interpolation and a much lower σ (0.005) value, and Figure A1C compares the histogram of FA values for the reference and registered test images using the cited interpolation methods. As can be seen from these images, using *sinc* interpolation and a lower σ the FA values gets closer to those from the original image.

#### Registration

We extended FSL to register DW-MR images, trying always to minimize the impact on the library, by using as much as possible the algorithms and classes already implemented there. Given that working with the whole DW-MR image with AI increases the computation cost, especially for nonlinear registration, we parallelized the linear and nonlinear registration algorithms using OpenMP^1^ and modify the AI algorithm to reduce computational costs. As can be seen from (3) and (18), we need to combine linearly hundreds of volumes, which is a costly operation. We reduced significantly the running time by fixing a low σ and using a kd-tree to search for the 16 closest gradients to a given target gradient orientation. The value of σ and the number of closest neighbors used were found experimentally as those values that produces a negligible difference with the original algorithm that uses all the volumes in the DW-MR image. The kd-tree is constructed only once, using all the gradients in the image, and it is used later in all the computations involving AI, which provides a reduction in the time complexity of the algorithm from *O*(*mn*) to *O*(*n log*(*m*)), where *m* is the number of volumes in the image and *n* the number of voxels.

We investigated also the effect of the number of volumes, for nonlinear registration, and found that using just *m* = 65 volumes were enough to register the whole DW-MRIs obtaining results very close to those using all the volumes in the original images (128 volumes), which provides a further reduction in the running time of nonlinear registration. Currently, linear registration can take 15 min using 4 processors and all the volumes in the image, while nonlinear registration can take 30–45 min using 8 processors and 65 volumes.

In MedINRIA, we used the b0 volume to obtain a transformation matrix and then perform linear and nonlinear registration of the estimated DT-MRIs. DTI-TK provides registration algorithms to register linearly and nonlinearly (Zhang et al., 2006) DT-MRIs as well as tools to convert from the tensor estimates from FSL-dtifit to the NIFTI^2^ format that DTI-TK adheres to.

**Table A1:** **John Hopkins University (JHU) white matter selected labels**.

Label	Region
1	Genu of corpus callosum
2	Body of corpus callosum
3	Medial lemniscus R
4	Medial lemniscus L
5	Inferior cerebellar peduncle R
6	Inferior cerebellar peduncle L
7	Superior cerebellar peduncle R
8	Superior cerebellar peduncle L
9	Cerebral peduncle R
10	Cerebral peduncle L
11	Anterior limb of internal capsule R
12	Anterior limb of internal capsule L
13	Posterior limb of internal capsule R
14	Posterior limb of internal capsule L
15	Retrolenticular part of internal capsule R
16	Retrolenticular part of internal capsule L
17	Anterior corona radiata R
18	Anterior corona radiata L
19	Superior corona radiata R
20	Superior corona radiata L
21	Posterior corona radiata R
22	Posterior corona radiata L
23	Posterior thalamic radiation (include optic radiation) R
24	Posterior thalamic radiation (include optic radiation) L
25	Sagittal stratum (include inferior longitidinal fasciculus and inferior fronto-occipital fasciculus) R
26	Sagittal stratum (include inferior longitidinal fasciculus and inferior fronto-occipital fasciculus) L
27	External capsule R
28	External capsule L
29	Cingulum (cingulate gyrus) R
30	Cingulum (cingulate gyrus) L
31	Cingulum (hippocampus) R
32	Cingulum (hippocampus) L
33	Fornix (cres) / Stria terminalis (can not be resolved with current resolution) R
34	Fornix (cres) / Stria terminalis (can not be resolved with current resolution) L
35	Superior longitudinal fasciculus R
36	Superior longitudinal fasciculus L
37	Superior fronto-occipital fasciculus (could be a part of anterior internal capsule) R
38	Superior fronto-occipital fasciculus (could be a part of anterior internal capsule) L
39	Uncinate fasciculus R
40	Uncinate fasciculus L

*The following regions were not considered, since they are located in the lower brain areas and that were not always fully visible in our datasets because of restricted field of view: middle cerebellar peduncle, pontine crossing tract, splenium of corpus callosum, fornix (column and body of fornix), corticospinal tract R, corticospinal tract L, tapetum R, and tapetum L*.

^1^http://openmp.org/wp. ^2^http://nifti.nimh.nih.gov/
